# Analysis of Relationships between Metabolic Changes and Selected Nutrient Intake in Women Environmentally Exposed to Arsenic

**DOI:** 10.3390/metabo14010075

**Published:** 2024-01-22

**Authors:** Monika Sijko-Szpańska, Lucyna Kozłowska

**Affiliations:** Laboratory of Human Metabolism Research, Department of Dietetics, Institute of Human Nutrition Sciences, Warsaw University of Life Sciences, 02776 Warsaw, Poland

**Keywords:** inorganic arsenic, metabolic pathways, diet, untargeted metabolomic, metabolism, exposure

## Abstract

Nutrients involved in the metabolism of inorganic arsenic (iAs) may play a crucial role in mitigating the adverse health effects associated with such exposure. Consequently, the objective of this study was to analyze the association between the intake levels of nutrients involved in iAs metabolism and alterations in the metabolic profile during arsenic exposure. The study cohort comprised environmentally exposed women: WL (lower total urinary arsenic (As), *n* = 73) and WH (higher As, *n* = 73). The analysis included urinary untargeted metabolomics (conducted via liquid chromatography–mass spectrometry) and the assessment of nutrient intake involved in iAs metabolism, specifically methionine, vitamins B_2_, B_6_, and B_12_, folate, and zinc (based on 3-day dietary records of food and beverages). In the WL group, the intake of all analyzed nutrients exhibited a negative correlation with 5 metabolites (argininosuccinic acid, 5-hydroxy-L-tryptophan, 11-trans-LTE4, mevalonic acid, aminoadipic acid), while in the WH group, it correlated with 10 metabolites (5-hydroxy-L-tryptophan, dihyroxy-1H-indole glucuronide I, 11-trans-LTE4, isovalerylglucuronide, 18-oxocortisol, 3-hydroxydecanedioic acid, S-3-oxodecanoyl cysteamine, L-arginine, p-cresol glucuronide, thromboxane B2). Furthermore, nutrient intake demonstrated a positive association with 3 metabolites in the WL group (inosine, deoxyuridine, glutamine) and the WH group (inosine, N-acetyl-L-aspartic acid, tetrahydrodeoxycorticosterone). Altering the intake of nutrients involved in iAs metabolism could be a pivotal factor in reducing the negative impact of arsenic exposure on the human body. This study underscores the significance of maintaining adequate nutrient intake, particularly in populations exposed to arsenic.

## 1. Introduction

During life, many factors such as biological, physical, and chemical may influence human health. One such factor is arsenic exposure, stemming from environmental pollution or occupational hazards. Environmental exposure encompasses contamination from both natural sources, such as volcanic activity and dust, and pollution tied to human activities like mining, smelting, and coal-powered plants. Water pollution, resulting from either natural or human-induced processes, adds to the reality that food can also serve as a source of environmental arsenic exposure [1,2].

The adverse impact of arsenic on the human body significantly depends on its chemical form. The International Agency for Research on Cancer classifies inorganic arsenic (iAs) compounds, including arsenic trioxide, arsenite, and arsenate, as Group 1 carcinogens to humans. On the other hand, organic compounds like dimethylarsinic acid and monomethylarsonic acid are categorized as Group 2B, possibly carcinogenic to humans, while arsenobetaine and other organic compounds fall into Group 3, not classifiable as to their carcinogenicity to humans. iAs compounds are linked to cancers of the lung, urinary bladder, and skin; additionally, a positive association exists between arsenic exposure and iAs compounds with cancers of the kidney, liver, and prostate [3]. Furthermore, prolonged arsenic exposure is linked to various other diseases, including cardiovascular issues [4], type 2 diabetes [5], chronic kidney disease [6], and neurodegenerative diseases [7]. Recent research has increasingly focused on the impact of arsenic exposure on women’s health. One study indicated that arsenic may disrupt the homeostasis of thyroid hormones, correlating with a negative relationship between its blood concentration and the levels of total triiodothyronine and thyroxine [8]. In another study by Liang et al. [9], a higher arsenic concentration in the blood was associated with the risk of developing polycystic ovarian syndrome and positively correlated with the concentrations of luteinizing hormone and the luteinizing hormone/follicle-stimulating hormone ratio. Moreover, arsenic exposure in pregnant women was linked to unfavorable birth outcomes in children, including lower birth weight and length [10].

The metabolism of iAs in the human body is intricate, involving multiple stages. iAs undergoes conversion to monomethylarsonic acid and subsequently to dimethylarsinic acid, with these forms predominantly excreted in urine. Within the iAs metabolism, one-carbon metabolism (OCM) plays a crucial role by providing the necessary S-adenosyl-methionine. The OCM process relies on nutrients such as methionine, betaine, choline, and folic acid (as methyl donors), along with vitamins B_2_, B_6_, B_12_, and zinc (serving as cofactors in reactions) [11,12,13].

Due to their involvement in iAs metabolism, these dietary compounds seem to play a crucial role in mitigating the adverse health effects associated with arsenic exposure. The positive impact of these nutrients on iAs metabolism has been partially explored. For instance, in the latest review, some studies propose that nutrients participating in iAs metabolism may affect methylation efficiency. However, the research findings are not entirely conclusive [14]. They mainly concentrate on the correlation between nutrient intake and the concentration of arsenic metabolites in urine or the risk of developing diseases. These studies do not delve into the detailed changes occurring in the body due to arsenic exposure and their connection to the consumption of specific nutrients. Therefore, our study aims to analyze the associations between alterations in the urinary metabolic profile and the level of nutritional intake involved in iAs metabolism in two groups of women environmentally exposed to arsenic, with lower vs. higher total urinary arsenic concentrations.

## 2. Materials and Methods

### 2.1. Study Participants

**Figure 1:**
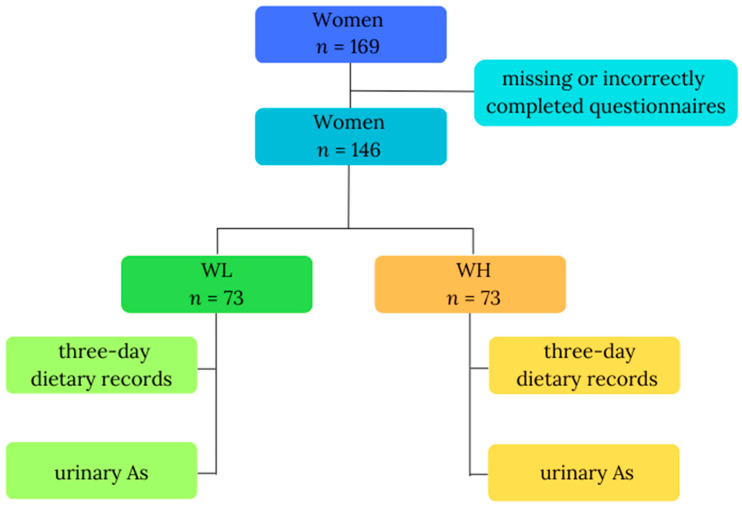
Study design. Abbreviations: As—total urinary arsenic; WL—the group of women with lower total urinary arsenic; WH—the group of women with higher total urinary arsenic.

This study was carried out among a cohort of women residing in the proximity of copper smelters, environmentally exposed to arsenic, probably mainly via inhalation. The previous study confirmed that inhabitants living in the vicinity of a copper smelter had a high total arsenic concentration in their urine [15]. Information was collected from all participants, encompassing a questionnaire with fundamental data (age, body weight, height), 3-day dietary records detailing consumed food and beverages, and a single urine sample. Data were initially gathered from 169 study participants; however, due to missing (*n* = 2; body mass) or incorrectly completed questionnaires (*n* = 21; inadequately filled 3-day dietary records), only data from 146 participants were included in the final analysis. The inclusion criteria comprised gender (female), residence in an industrial copper region in Poland, 18 to 85 years of age, nonpregnant women, and everyone who volunteered to participate and signed informed consent. Participants were then divided into two groups based on their total urinary arsenic (As) concentrations: WL, denoting the group of women with lower As (*n* = 73), and WH, representing the group of women with higher As (*n* = 73). Figure 1 illustrates the study design. All participants willingly and knowingly provided their consent for involvement. We used methods for keeping data confidential, such as substituting codes for participant identifiers and storing data in locked cabinets; files containing electronic data were password-protected and access to code lists or key codes was limited. The study received ethical approval from the Ethics Committee of the Nofer Institute of Occupational Medicine in Lodz, Poland, with reference number 08/2020, and all participants signed the informed consent form.

### 2.2. Urine Collection and As Analysis

Urine samples collected between October 2021 and December 2022 were stored at −80 °C until subjected to metabolomics analyses. To determine the concentration of As, an ELAN DRC-e Inductively Coupled Plasma Mass Spectrometer (ICP-MS), equipped with a Dynamic Reaction Cell (Perkin Elmer, SCIEX, Waltham, MA, USA), was used, following a protocol previously published [16].

### 2.3. Diet Assessment

The information gleaned from the 3-day dietary records of food and beverages underwent meticulous analysis using Dieta 6.0 software, specifically crafted by the National Institute of Food and Nutrition in Warsaw, Poland, for assessing the dietary intake of selected nutrients. A certified dietitian utilized the “Album of Product and Dish Photographs” [17] to precisely determine and clarify portion sizes, aligning with international nutritional research standards. Ensuring accuracy in reporting dietary habits is paramount, with errors like underreporting and overreporting of consumption potentially introducing discrepancies. To identify atypical data and errors and assess data reliability, we employed the Goldberg method [18]. In line with this method, data from days with dietary energy values lower than the individual’s basal metabolic rate (using a cut-off point of 0.76) were excluded from the analysis, with the basal metabolic rate calculated using the Harris–Benedict equation. The dietary intake of selected nutrients is presented as the mean/median intake over 3 days and, additionally, as the mean/median intake per kilogram of body mass. Contextualizing this information, the intake of nutrients involved in iAs metabolism was compared to nutrition norms for the Polish population at the level of the estimated average requirement (EAR) [19].

### 2.4. Urine Sample Preparations and Untargeted Metabolomics Analysis

Urine samples were prepared following the protocol outlined by Southam et al. [20]. The samples were randomized, divided into three batches, and subjected to analysis using two distinct assays. Assay 1 was employed for the extraction of nonpolar and semipolar metabolites, while assay 2 was utilized for extracting polar metabolites. The procedural details for both assays are illustrated in Figure S1 in the Supplementary Materials.

Quality control (QC) samples were meticulously prepared by combining equal volumes of aliquots (100 μL) from every urine sample. These QC samples played a crucial role in monitoring system stability through regular injections, conducted after every 10 experimental samples. Each batch encompassed samples for system equilibration (10), subject samples (58), QC samples (8), and blank samples (2). Details regarding the reagents used for the metabolomics analysis can be found in the Supplementary Materials (Table S1).

For the urine metabolomics analysis, we utilized the Waters AcquityTM Ultra Performance LC system (Waters Corp., Milford, MA, USA) connected to a Synapt G2Si Q-TOF mass spectrometer (Waters MS Technologies, Manchester, UK), equipped with an electrospray (ESI) source (Waters, Manchester, UK). Metabolite separation was executed using an ACQUITY UPLC HSS T3 precolumn (1.8 µm, VanGuard Precolumn 2.1 × 5 mm) connected with an ACQUITY UPLC HSS T3 chromatography column (1.8 µm, 2.1 × 100 mm) for assay 1, and an ACQUITY UPLC BEH Amide precolumn (1.7 µm, VanGuard Precolumn 2.1 × 5 mm) connected with an ACQUITY UPLC BEH Amide (1.7 µm, 2.1 × 100 mm) chromatography column (Waters, Milford, MA, USA) for assay 2. Analyses were conducted in positive and negative ionization modes, with detailed information available in a previous study [21]. Chromatographic separation parameters for assay 1 included a sample injection volume of 4 µL, mobile phases A (ultra-high purity water and 0.1% formic acid) and B (acetonitrile and 0.1% formic acid). The flow gradient used was: 0–2 min 99% phase A and 1% phase B; 2–4 min 90% A and 10% B; 4–5 min 80% A and 20% B; 5–6 min 70% A and 30% B; 6–8 min 50% A and 50% B; 8–11.5 min 1% A and 99% B; 11.5–15 min 99% A and 1% B. For assay 2, the sample injection volume was 2.5 µL, with mobile phases A (95% acetonitrile, 5% ultra-high purity water, 10 mM ammonium formate, and 0.1% formic acid) and B (50% acetonitrile, 50% ultra-high purity water, 10 mM ammonium formate, and 0.1% formic acid). The flow gradient in positive ionization mode was: 0–3 min 99% phase A and 1% phase B; 3–6 min 88% A and 12% B; 6–8 min 50% A and 50% B; 8–9 min 30% A and 70% B; 9–11.5 min 1% A and 99% B; 11.5–15 min 99% A and 1% B. In negative ion mode: 0–3 min 99% phase A and 1% phase B; 3–6 min 85% A and 15% B; 6–9 min 30% A and 70% B; 9–10 min 5% A and 95% B; 10–10.5 min 1% A and 99% B; 10.5–15 min 99% A and 1% B. The chemical reagents and mass spectrometer parameters were consistent with previous descriptions [21]. A fast data-dependent acquisition method was employed for compound fragmentations, utilizing the same parameters as in the metabolomic analysis. The Human Metabolome Database (HMDB) [22] was used for comparing resulting fragmentation spectra to putatively annotate compounds.

### 2.5. Data Processing and Statistical Analysis

For data processing, involving feature detection, retention time correction, alignment, and putative annotation of compound classes, the collected files were imported into Progenesis QI v3.0 software (Waters, Milford, MA, USA). Subsequently, the data matrices underwent filtration, removing features with a QC relative standard deviation greater than 40% (for positive ionization) and greater than 35% (for negative ionization) in each batch separately. Additionally, features with a blank contribution greater than 5% and missing values exceeding 60% in a table with all features were excluded. Normalization was carried out on the online platform MetaboGroupS (https://www.omicsolution.com/wukong/MetaboGroupS/ accessed on 29 June 2023) [23] to mitigate unwanted variations, such as signal drift and batch effects. The EigenMs normalization method was chosen as the most appropriate, based on the minimum coefficients of variation.

A peak intensity table comprising 9484 features from assays 1 and 2, as well as from positive and negative ionization modes, was utilized for statistical analysis in MetaboAnalyst (https://www.metaboanalyst.ca/home.xhtml accessed on 5 July 2023) [24]. In the fold change analysis (fold change threshold 1.1 and 0.9), 1720 features exhibited differences between the WL and WH groups. Features above 1.1 and below 0.9 fold change were further considered for analysis, resulting in 942 features. These features were then incorporated into the analysis of correlations with the intake of nutrients involved in iAs metabolism. From the features that correlated with As in the entire group of women, only those also correlating with nutrient intake were selected for annotation.

Statistical analyses were performed using Statistica software, version 13.0 (StatSoft Inc., Tulsa, OK, USA). The normality of data distribution was assessed using the Shapiro–Wilk test. For comparing variables between two groups, the Student’s *t*-test was employed for parametric distributions, and the Mann–Whitney U test was used for nonparametric distributions. Correlation analyses between the intake of nutrients involved in iAs metabolism and putatively annotated metabolites were conducted using the Pearson correlation coefficient or the Spearman rank correlation coefficient. The value of the assumed level of statistical significance was *p* ≤ 0.05. To identify potential biomarkers, classical univariate receiver operating characteristic (ROC) curve analysis was performed in MetaboAnalyst in the Biomarker Analysis module (https://www.metaboanalyst.ca/home.xhtml accessed on 29 September 2023).

## 3. Results

### 3.1. General Characteristic

The general characteristics of the group of women are summarized in Table 1.

No significant differences were observed between the WL and WH groups concerning age, height, and body mass. However, higher values of As were noted in the WH group compared to WL group (Table 1).

Table 2 presents the mean/median daily intake per kilogram of body mass and Table S2 (in Supplementary Materials) per day of nutrients involved in iAs metabolism, including: methionine; vitamins B_2_, B_6_, and B_12_; folate; and zinc.

Significant differences were noted in the intake of vitamin B_6_, both in mg/day and in mg/kg body mass, between the WL and WH groups; the intake of other analyzed nutrients did not show statistical differences. The intake of almost all analyzed nutrients was above the EAR, except for folate. In both the WL and WH groups, the mean dietary intake of folate was lower than the EAR (Table S2).

### 3.2. Metabolic Differences between WL and WH Groups and Their Relationship with As

**Figure 2:**
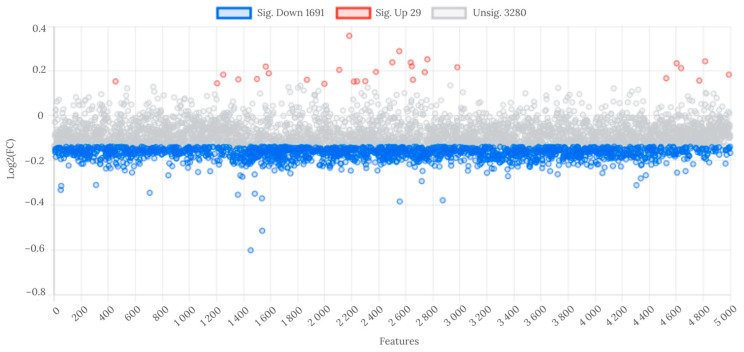
Analysis of fold change in metabolite features between WL and WH groups. Abbreviations: WL—the group of women with lower total urinary arsenic; WH—the group of women with higher total urinary arsenic.

At the fold change threshold of ≥1.1 and ≤0.9, 29 features were significantly upregulated, and 1691 were downregulated in WL group compared to WH group (Figure 2).

Only putatively annotated endogenous metabolites, identified as exposure effect metabolites, that correlated with As (Table 3) and the intake of selected nutrients involved in iAs metabolism were included in the subsequent description. In total, we putatively annotated 41 metabolites, comprising 18 endogenous and 23 exogenous (as food ingredients). The supplementary materials include a detailed table (Table S3) listing all putatively annotated metabolites.

Almost all putatively annotated metabolites exhibited a positive correlation with As in the entire group, except for inosine, which showed a negative correlation. Similar findings were observed in the signal intensity of 18 putatively annotated metabolites, significantly higher in the WH group compared to WL group, with the exception of one metabolite, inosine, where a lower signal intensity was noted in the WH group (Table S2).

### 3.3. Association between Dietary Nutrient Intake Involved in iAs Metabolism and Signal Intensity of Putatively Annotated Metabolites

#### 3.3.1. Negative Association

A correlation analysis was conducted between the dietary intake of nutrients involved in iAs metabolism and the signal intensity of putatively annotated metabolites. This analysis was performed on the entire group of women, as well as separately in the WL and WH groups.

A negative association was observed between the intake of the analyzed nutrients and the signal intensity of thirteen putatively annotated metabolites (Table 4). These metabolites belong to six pathways: carbohydrate metabolism (dihyroxy-1H-indole glucuronide I, isovalerylglucuronide), lipid metabolism (11-trans-LTE4, 18-oxocortisol, 3-hydroxydecanedioic acid, S-3-oxodecanoyl cysteamine, thromboxane B2), amino acid metabolism (argininosuccinic acid, 5-hydroxy-L-tryptophan, aminoadipic acid, L-arginine, p-cresol glucuronide), and metabolism of terpenoids and polyketides (mevalonic acid).

In both groups, negative associations were observed between the intake of methionine and zinc and two metabolites: 11-trans-LTE4 and L-arginine.

In the WL group, the intake of all analyzed nutrients (methionine, vitamins B_2_, B_6_ and B_12_, folate, and zinc) was negatively correlated with five metabolites: argininosuccinic acid, 5-hydroxy-L-tryptophan, 11-trans-LTE4, mevalonic acid, and aminoadipic acid.

In the WH group, negative associations were observed between the intake of all the analyzed nutrients and ten metabolites: 5-hydroxy-L-tryptophan, dihyroxy-1H-indole glucuronide I, 11-trans-LTE4, isovalerylglucuronide, 18-oxocortisol, 3-hydroxydecanedioic acid, S-3-oxodecanoyl cysteamine, L-arginine, p-cresol glucuronide, and thromboxane B2.

Notably, only 11-trans-LTE4 showed correlation with zinc intake in both the WL and WH groups.

#### 3.3.2. Positive Association

Positive correlations were observed between the intake of all analyzed nutrients and five putatively annotated metabolites belonging to three pathways (Table 5). These pathways include lipid metabolism (tetrahydrodeoxycorticosterone), nucleotide metabolism (inosine, deoxyuridine), and amino acid metabolism (glutamine, N-acetyl-L-aspartic acid).

In both groups, the intake of all the analyzed nutrients (methionine, vitamins B_2_, B_6_ and B_12_, folate, and zinc) was positively associated with four metabolites: inosine, N-acetyl-L-aspartic acid, deoxyuridine, and glutamine.

In the WL group, the intake of all analyzed nutrients was also positively correlated with three metabolites: inosine, deoxyuridine, and glutamine.

In the WH group, positive correlations were observed between the intake of all the analyzed nutrients and three metabolites: inosine, N-acetyl-L-aspartic acid, and tetrahydrodeoxycorticosterone.

In both the WL and WH groups, inosine was positively associated with the intake of vitamin B_2_.

### 3.4. Biomarker Analysis

**Figure 3:**
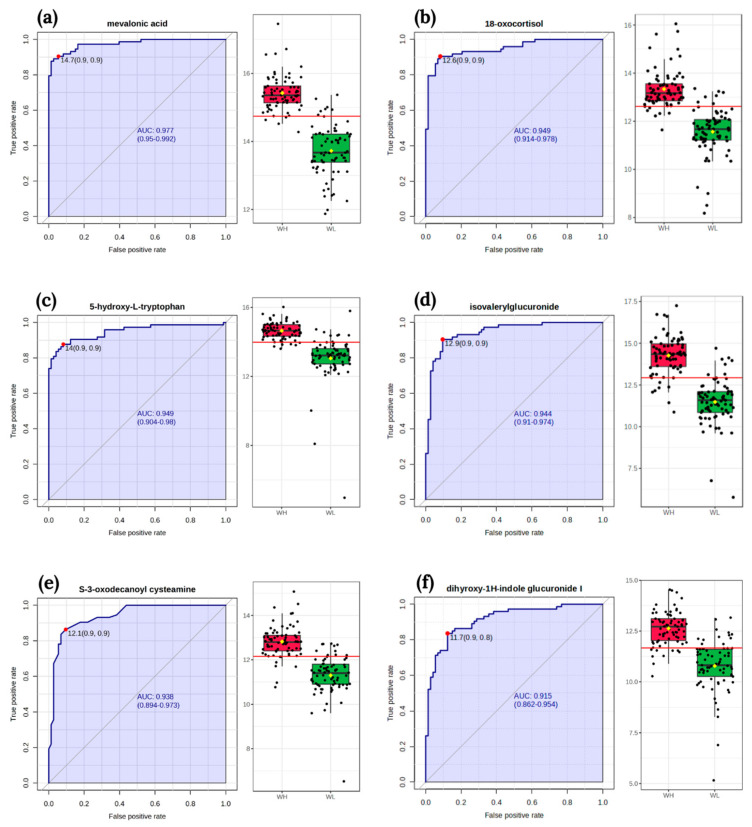
The receiver operating characteristic curves of biomarker and box-plot of the signal intensity of the metabolites between two groups WL and WH: (**a**) mevalonic acid; (**b**) 18-oxocortisol; (**c**) 5-hydroxy-L-tryptophan; (**d**) isovalerylglucuronide; (**e**) S-3-oxodecanoyl cysteamine; (**f**) dihyroxy-1H-indole glucuronide I. The sensitivity is on the y-axis, and the specificity is on the x-axis. The area under the curve is in blue. Abbreviations: WL—the group of women with lower total urinary arsenic; WH—the group of women with higher total urinary arsenic.

The classical univariate ROC curve analysis revealed six metabolites (mevalonic acid, 18-oxocortisol, 5-hydroxy-L-tryptophan, isovalerylglucuronide, S-3-oxodecanoyl cysteamine, dihyroxy-1H-indole glucuronide I) as potential biomarkers of arsenic exposure. The area under the curve (AUC) value for these metabolites exceeded 0.9 (Figure 3).

## 4. Discussion

To the best of our knowledge, this is the first study conducted in a group of women in the general population to analyze the intake of nutrients involved in iAs metabolism and its association with changes in body metabolism using an untargeted metabolomics method. The study results revealed several relationships between nutrient intake and changes in metabolic pathways, including carbohydrate, lipid, amino acid, terpenoids and polyketides, and nucleotide metabolism. The primary focus is on putatively annotated metabolites that correlated with the intake of nutrients involved in iAs metabolism.

### 4.1. Urinary As Concentrations

In our study, the median As concentration in the entire group of women was 5.0 μg/L (range: 0.1–339.5 μg/L). In studies conducted by other authors, the As concentrations were higher in different groups of women and were as follows: 65.0 μg/L (range: 12.0–407.0 μg/L, indigenous women around Lake Poopó, Bolivia) [25]; 17.3 μg/L (range: 0.4–641.4 μg/L, postmenopausal Danish women) [26]; and 29.6 μg/L (range: 18.9–46.7 μg/L, pregnant women, China) [27]. The As concentration is influenced by various factors, including the level and source of arsenic exposure, ethnicity, age, sex, pregnancy, smoking, and nutrition. Some factors affect arsenic metabolism and contribute to variations in urinary arsenic species. For instance, women have better methylation capacity than men, and it also increases during pregnancy. The age effect on arsenic metabolism needs to be confirmed; it has been suggested that decreasing methylation capacity is associated with increasing age [28].

The reference value (RV 95) for As in children and adults (who did not consume fish within 48 h prior to sample collection) is set at 15 μg/L [29]. In our study, approximately 20% of all women and nearly 40% in the WH group had As concentrations exceeding this threshold.

### 4.2. Metabolites That Were Negatively Associated with Nutrient Intake

Due to the lack of similar studies between nutrient intake and metabolites’ signal intensity, we focused on describing metabolites and their association with negative health effects. These negative effects may be reduced by a higher intake of nutrients involved in iAs metabolism, as indicated by the negative relationships between the signal intensity of the described metabolites and the intake of nutrients involved in iAs metabolism.

#### 4.2.1. Carbohydrate Metabolism

In the carbohydrate metabolism pathway, we identified two metabolites: dihyroxy-1H-indole glucuronide I and isovalerylglucuronide. In metabolomics studies involving humans exposed to various chemicals, no similar results to those observed with dihyroxy-1H-indole glucuronide have been reported. However, analogous findings were demonstrated in one study involving an animal model. In a group of mice exposed to iAs, an increased serum signal intensity of dihyroxy-1H-indole glucuronide was observed [30]. Li et al. [31] classified dihyroxy-1H-indole glucuronide I as a potential marker of breast cancer, as a higher signal intensity of this metabolite was noted in breast cancer samples compared to controls. Numerous studies have explored the relationship between arsenic exposure and the risk of developing breast cancer, highlighting As as one of the risk factors [32,33].

In Bangladeshi adults exposed to arsenic from drinking water, 3-hydroxyisovaleric acid was identified in the urine, showing a negative correlation with As (borderline of significance, *p* = 0.052) [34]. Hine and Tanaka [35] observed in patients with isovaleric acidemia that isovalerylglucuronide is more likely to be excreted when the amount of 3-hydroxyisovaleric acid excreted in the urine is high. 3-hydroxyisovaleric acid may be implicated in several diseases, for instance, it could serve as one of the prognostic markers for ovarian carcinomas [36]. An increased concentration of this metabolite was also observed in diabetic patients [37].

#### 4.2.2. Lipid Metabolism

We also putatively annotated several metabolites involved in lipid metabolism: 11-trans-LTE4, 18-oxocortisol, 3-hydroxydecanedioic acid, S-3-oxodecanoyl cysteamine, and thromboxane B2.

11-trans-LTE4 is an isomer of LTE4. In line with our findings in the general population of women, Li et al. [38] putatively identified two LTE4 metabolites, 18-carboxy-dinor-LTE4 and 20-COOH-LTE4, in the urine of Chinese pregnant women exposed to arsenic. These two metabolites serve as oxidation indicators, and their urine signal intensity was higher in the group of women with higher arsenic exposure. Lopez-Vicario et al. [39] reported higher concentrations of LTE4 in the serum associated with acute-on-chronic liver failure (ACLF). LTE4 concentrations increased with the development of the disease, and positively correlated with Keratin 18 (a marker of cell death) and Interleukin-8 (an inflammatory cytokine). Increased concentrations of urinary LTE4 were also observed in patients with hepatorenal syndrome [40] and systemic mastocytosis [41].

Cortisol can undergo conversion to 18-hydroxycortisol and subsequently to 18-oxocortisol [42]. The plasma signal intensity of cortisol was found to be negatively associated with As in diabetic patients residing in the chronic arsenic exposure region of Chihuahua, Mexico [43], suggesting the potential conversion of cortisol during arsenic exposure. Additionally, in an animal model study involving mice exposed to ambient PM2.5 (which may contain arsenic), the signal intensity of two stress hormone metabolites in the serum (18-oxocortisol and 5a-tetrahydrocortisol) was higher than in the control group [44].

To the best of our knowledge, there are no untargeted metabolomics studies demonstrating a connection between exposure to various compounds and changes in the signal intensity of 3-hydroxydecanedioic acid (also known as 3-hydroxysebacic acid). This metabolite may be linked to mental illness. In a serum metabolomics study, 3-hydroxysebacic acid was identified as one of the potential biomarkers of anxiety disorders [45]. In a rat model of Alzheimer’s disease, an increased signal intensity of 3-hydroxysebacic acid in hair was also observed [46]. Arsenic exposure has been associated with a higher risk of generalized anxiety disorder [47] and may also be linked to neurodegenerative diseases and cognitive impairment [7].

In the existing literature, there is a lack of metabolomics studies specifically related to arsenic exposure or other compounds. However, there are studies where the intensity of S-3-oxodecanoyl cysteamine changes in the context of various diseases. For instance, in patients with type 1 diabetes mellitus, urinary S-3-oxodecanoyl cysteamine was upregulated in the progressive group compared to the nonprogressive normoalbuminuric group [48]. In a mouse model exposed to a high dose of ergotamine (an ergot alkaloid used as a pharmaceutical agent), S-3-oxodecanoyl cysteamine in the cerebral cortex was upregulated compared to the control group. The authors suggested that this metabolite is related to energy metabolism, and disturbances in this pathway are a consequence of stress and toxicity [49].

In a serum metabolome study on rats exposed to ambient ozone (an air pollutant), thromboxane B2 (TXB2) was also upregulated [50]. The concentration of TXB2 was also higher in the group of children exposed to lead compared to the control group, and it was additionally positively associated with lead concentration in the blood [51]. In the THP-1 macrophages exposed to lead, an increased TXB2 concentration compared to controls was also observed. In this study, under lead exposure, there was also an increased expression of cyclooxygenase 1 and 2, which are proinflammatory enzymes. TXB2 may be a product of these enzymes [52]. A higher concentration of TXB2 in the serum was found in hyperthyroid patients than in euthyroid individuals. Thromboxane A2 (TXA2) can be converted to TXB2, and TXA2 is associated with pulmonary hypertension linked to hyperthyroidism [53].

#### 4.2.3. Amino Acid Metabolism

Regarding amino acid metabolism, several metabolites were annotated, including argininosuccinic acid, 5-hydroxy-L-tryptophan, aminoadipic acid, L-arginine, and p-cresol glucuronide, all of which were linked to nutrient intake.

In a urine metabolomics study in a group of women with higher As, an increased signal intensity of argininosuccinic acid was also observed [15]. Argininosuccinic acid may also serve as a potential biomarker of occupational exposure to hexavalent chromium, as indicated by increased urinary signal intensity in the worker group [21]. According to Xu et al. [54], argininosuccinic acid might be a potential biomarker for nonsmall cell lung cancer (NSCL). Additionally, argininosuccinic acid has been associated with toxicity for astrocytes and neurons [55], leading to increased lipid and protein oxidation, oxidative stress, and decreased antioxidant defense in the cerebral cortex of rats [56].

5-hydroxy-L-tryptophan is a metabolite of L-tryptophan. In a Chinese population exposed to arsenic, the signal intensity of tryptophan in the serum was negatively associated with arsenic-induced skin lesions (AISL), and a higher level of tryptophan showed the lowest odds of AISL [57]. iAs exposure in MIN6-K8 cells decreased the signal intensity of serotonin and its precursor, 5-hydroxy-L-tryptophan, along with a decrease in glucose-stimulated insulin secretion (GSIS). The authors demonstrated that iAs exposure may influence GSIS and involves serotonin metabolism. Moreover, iAs exposure increased mRNA expression of polypeptide a6a, the gene that codes a UDP-glucuronosyltransferase, an enzyme of the glucuronidation pathway. Glucuronidation facilitates the disposal of serotonin [58]. These studies indicate that arsenic exposure may disrupt tryptophan and serotonin metabolism, and 5-hydroxy-L-tryptophan is involved in both of these subpathways.

In two groups of female mice exposed to iAs, the signal intensity of urinary aminoadipic acid in the wild-type mice group was higher compared to the As3mt-knockout mice group [59]. Aminoadipic acid is a product of lysine degradation. In endothelial cells exposed to high glucose for 7 days, an increased aminoadipate level was observed, associated with lysine breakdown through oxidative stress [60]. Aminoadipic acid can also serve as a biomarker for diabetes risk [61] and a marker for protein oxidation [62].

Regarding L-arginine, a similar finding was reported in a urinary metabolomics study, but in a group of workers exposed to hexavalent chromium. A higher signal intensity of L-arginine in the exposed worker group compared to the control group was observed [21]. In turn, the concentration of L-arginine was decreased in the human vein endothelial cells exposed to iAs (5 μM for 24 h) but not in lower-level exposures (cells exposed to 1 or 2.5 μM). In this study, a decreased level of nitric oxide and dimethylarginine dimethylaminohydrolase (DDAH) 1 protein expression, and an increased concentration of asymmetric dimethylarginine (ADMA) were also observed. The authors discussed that under iAs exposure, L-arginine, ADMA, and DDAH are associated with nitric oxide depletion, related to oxidative stress [63].

Our results align with another study in which the signal intensity of p-cresol glucuronide was higher in the urine of pregnant women with high arsenic exposure than in the low arsenic exposure group [38]. This outcome contrasts with Huang et al. [64], who demonstrated a decreased signal intensity of p-cresol glucuronide in the kidneys of mice exposed to arsenic for 14 months compared to the control group. However, this metabolite was not annotated in the urine metabolome. Another interesting finding is that the p-cresol glucuronide concentration increased with chronic kidney disease (CKD) development (highest in stage 5 CKD) and was associated with an increased risk of total and cardiovascular mortality [65]. The relationship between arsenic exposure and CKD has been discussed in many studies [6,66].

The last putatively annotated metabolite, mevalonic acid, is involved in the metabolism of the terpenoids and polyketides pathway. Mevalonic acid is formed from 3-hydroxy-3-methylglutaryl-coenzyme A (HMG-CoA) with the participation of the HMG-CoA reductase enzyme, and in further stages, it can be converted to cholesterol or other compounds such as haem A, ubiquinone, or proteins [67]. Moreover, mevalonic acid in the urine positively correlates with cholesterol biosynthesis [68]. In the study by Qu and Huang [69], As was positively correlated with total cholesterol and low-density lipoprotein cholesterol concentration in the serum.

**Figure 4:**
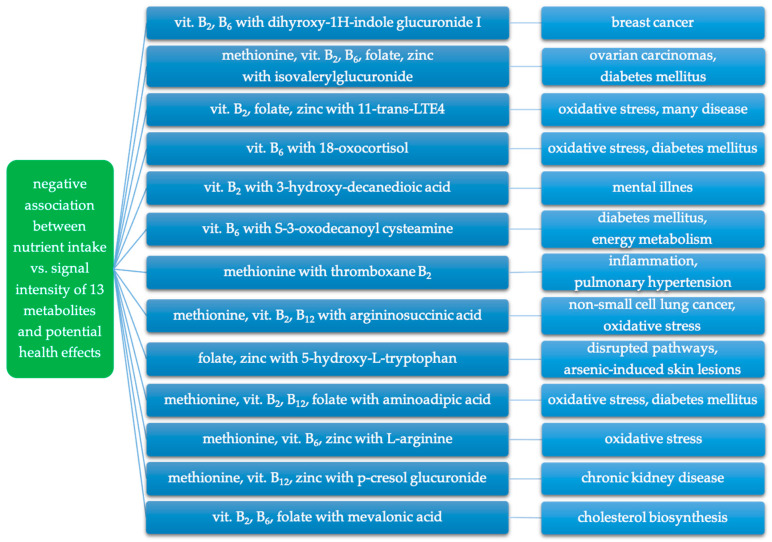
Negative association between intake of nutrients involved in iAs metabolism and putatively annotated metabolites and related potential adverse health effects.

Considering the correlations observed in our study and the findings of other studies regarding the described metabolites, it seems that a higher intake of methionine, vitamins B_2_, B_6_, B_12_, folate, and zinc may play a crucial role in modulating changes associated with these metabolites. A summary of the negative relationships between the intake of nutrients involved in iAs metabolism and the signal intensity of putatively annotated metabolites, and their potential adverse health effects is presented in Figure 4.

### 4.3. Metabolites That Were Positively Associated with Nutrient Intake

Our study also revealed positive associations between tetrahydrodeoxycorticosterone, inosine, deoxyuridine, N-acetyl-L-aspartic acid, glutamine, and the intake of nutrients involved in iAs metabolism.

Tetrahydrodeoxycorticosterone (THDOC) is an endogenous neurosteroid that may be elevated under stress and can modulate GABA receptors, having a sedative, anxiolytic, and anticonvulsant effect [70,71]. In a rat model on cultured hypothalamic neurons, an interaction between THDOC and GABA was observed, which was connected with a decrease or increase in the excitability of neurons [72].

In nucleotide metabolism, significant changes were observed in inosine and deoxyuridine. Inosine is a purine nucleoside, and its beneficial functions in the body have led to its use in the treatment of many diseases such as cardiovascular, infectious, neuropsychological, and cancers [73]. In contrast to our results, an increase in inosine concentration in the serum of mice was observed due to exposure to iAs [30].

Similarly, deoxyuridine, as a pyrimidine nucleotide, has been widely studied in neurodegenerative diseases. In a study on an Aβ25-35-induced mice model, 4-week administration of deoxyuridine, among other effects, reduced mitochondrial damage in the hippocampus and oxidative stress in the serum and brain, as well as apoptosis in primary mice hippocampal cells [74]. Another study showed that exposure to iAs influences the biosynthesis of nucleotides, and an increased level of deoxyuridine in human bronchial epithelial cells BEAS-2B was observed [75].

Glutamine and N-acetyl-L-aspartic acid belong to amino acid metabolism. Glutamine is involved in numerous processes; it is an endogenous amino acid but can also be obtained through the diet or via supplementation. Several studies have scrutinized the impact of glutamine supplementation on cardiometabolic risk factors, showing reductions in fasting plasma glucose and C-reactive protein [76]. Additionally, in colorectal cancer, glutamine supplementation has been associated with decreased tumor necrosis factor-α, a reduced risk of wound infections, and shorter hospital stays [77]. In a study by Pai et al. [78] on mice exposed to iAs, glutamine supplementation reduced the expression of leukocyte integrin, specifically decreasing leukocyte-function-associated antigen-1 and macrophage antigen-1 in the blood, mitigating the inflammatory response.

In a mouse model exposed to cadmium, a higher signal intensity of N-acetyl-L-aspartic acid in the urine was observed [79], mirroring our results. Conversely, decreased concentrations of N-acetyl-L-aspartic acid in several cerebral regions have been noted in Alzheimer’s disease [80] and schizophrenia [81]. Much research has delved into environmental toxicants, including arsenic exposure, and their impact on the development of neurodegenerative diseases [82].

The positive relationships we observed between tetrahydrodeoxycorticosterone, deoxyuridine, inosine, N-acetyl-L-aspartic acid, glutamine, and the intake of methionine, vitamins B_2_, B_6_, B_12_, folate, and zinc may indirectly enhance the aforementioned effects and may indicate a beneficial effect of these nutrients on many pathways. However, the availability of results in this area is very limited, which does not allow for an in-depth discussion and explanation of our findings.

### 4.4. Strengths and Limitations

The strengths and limitations of our study need to be highlighted. This marks the inaugural investigation into a cohort of environmentally arsenic-exposed women wherein data on nutrient intake relevant to iAs metabolism were amalgamated with metabolic analysis results. Consequently, the study offers comprehensive insights into the metabolic profile of this specific cohort and its interplay with nutrient intake. Furthermore, the findings indicate that arsenic exposure correlates with alterations in multiple metabolic pathways, emphasizing a broader focus beyond the analysis of arsenic metabolites in urine. Another robust aspect of the study involves the meticulous determination of As concentration using well-established methods and considering the intake of various nutrients. Nonetheless, our results are not exempt from limitations. Generalizing the study outcomes to the wider population is constrained as only women were included. The study solely gauged the concentration of As, without speciation considerations, and relied on a single urine sample. Additional limitations pertain to nutritional assessment, where despite efforts to mitigate errors, the use of 3-day dietary records may not fully capture factors like seasonal variations in intake. In summation, while acknowledging these limitations, our results should be viewed preliminarily. Despite the constraints, the data offer promising indications that dietary modifications might mitigate the adverse effects of arsenic exposure in the human body.

## 5. Conclusions

In two groups of women environmentally exposed to arsenic (WH vs. WL), numerous distinctions in the metabolic profile were evident. Putatively annotated endogenous metabolites were predominantly associated with amino acid, carbohydrate, and lipid metabolism, with their signals intensifying notably more in WH group compared to WL group. Both negative and positive correlations emerged between the intake of methionine, vitamins B_2_, B_6_, and B_12_, folate, and zinc and the signal intensity of the putatively annotated metabolites in the WL and WH groups. Concerning negative correlations, it appears that a higher nutrient intake might alleviate the adverse impact of arsenic exposure. However, additional explanations are warranted for positive correlations. These findings underscore the pivotal role of nutrient intake in mitigating the adverse effects of arsenic, underscoring the significance of dietary interventions, particularly in arsenic-exposed populations.

## Figures and Tables

**Table 1:** General characteristics of the women environmentally exposed to arsenic.

Parameter	Both Groups		WL		WH		*p*-Value **
	*n* = 146		*n* = 73		*n* = 73		
	Mean ± SD	Median (Minimum–Maximum)	Mean ± SD	Median (Minimum–Maximum)	Mean ± SD	Median (Minimum–Maximum)	
Age (years)	59.2 ± 14.4	62.5 (18.0–85.0)	60.3 ± 15.8	65.0 (18.0–84.0)	58.1 ± 12.9 *	59.0 (23.0–85.0)	0.1021
Height (cm)	163.5 ± 6.5 *	163.0 (147.0–184.0)	163.0 ± 6.2 *	163.0 (152.0–176.0)	164.0 ± 6.7 *	164.0 (147.0–184.0)	0.3659
Body mass (kg)	69.5 ± 12.6	67.5 (47.0–108.0)	68.3 ± 12.0	67.0 (48.0–108.0)	70.7 ± 13.0 *	70.0 (47.0–100.0)	0.2669
As (µg/L)	14.0 ± 34.8	5.0 (0.1–339.5)	2.5 ± 1.2 *	2.3 (0.1–5.0)	25.6 ± 46.5	10.8 (5.0–339.5)	0.0000

Abbreviations: *—parametric distribution data (used Shapiro–Wilk test *p* ≤ 0.05); **—the Student’s *t*-test (for parametric distribution) and Mann–Whitney U test (for nonparametric distribution) were used to examine differences between WL and WH; As—total urinary arsenic; WL—the group of women with lower total urinary arsenic; WH—the group of women with higher total urinary arsenic.

**Table 2:** Dietary intake of selected nutrients involved in iAs metabolism.

Dietary Intake	Both Groups		WL		WH		*p*-Value **
	*n* = 146		*n* = 73		*n* = 73		
	Mean ± SD	Median (Minimum–Maximum)	Mean ± SD	Median (Minimum–Maximum)	Mean ± SD	Median (Minimum–Maximum)	
Methionine (mg/kg bm)	22.14 ± 7.84	21.25 (7.37–60.83)	21.68 ± 6.74 *	21.64 (7.37–39.57)	22.61 ± 8.82	20.34 (9.22–60.83)	0.9003
Vitamin B_2_ (mg/kg bm)	0.02 ± 0.01	0.02 (0.01–0.04)	0.02 ± 0.01	0.02 (0.01–0.04)	0.02 ± 0.01 *	0.02 (0.01–0.04)	0.3298
Vitamin B_6_ (mg/kg bm)	0.02 ± 0.01	0.02 (0.01–0.06)	0.02 ± 0.01 *	0.02 (0.01–0.05)	0.03 ± 0.01	0.02 (0.01–0.06)	0.0239
Vitamin B_12_ (µg/kg bm)	0.03 ± 0.02	0.03 (0.01–0.15)	0.03 ± 0.01	0.03 (0.01–0.08)	0.04 ± 0.02	0.03 (0.01–0.15)	0.6302
Folate (µg/kg bm)	3.87 ± 1.55	3.63 (0.78–11.24)	3.75 ± 1.46	3.52 (1.62–10.03)	3.99 ± 1.63	3.93 (0.78–11.24)	0.2906
Zinc (mg/kg bm)	0.12 ± 0.04	0.12 (0.05–0.20)	0.12 ± 0.04 *	0.12 (0.05–0.20)	0.12 ± 0.04 *	0.12 (0.06–0.20)	0.8613

Abbreviations: *—parametric distribution data (used Shapiro–Wilk test *p* ≤ 0.05); **—the Student’s *t*-test (for parametric distribution) and Mann–Whitney U test (for nonparametric distribution) were used to examine differences between WL and WH; bm—body mass; EAR—estimated average requirement for Polish population; WL—the group of women with lower total urinary arsenic; WH—the group of women with higher total urinary arsenic.

**Table 3:** Correlations between As and signal intensity of endogenous metabolites in both groups of women (*n* = 146).

Metabolites	HMDB ID	R *	*p*-Value
isovalerylglucuronide	HMDB0002091	0.6334	0.0000
argininosuccinic acid	HMDB0000052	0.5867	0.0000
5-hydroxy-L-tryptophan	HMDB0000472	0.6017	0.0000
mevalonic acid	HMDB0000227	0.7124	0.0000
inosine	HMDB0000195	−0.2082	0.0117
18-oxocortisol	HMDB0000332	0.6790	0.0000
N-acetyl-L-aspartic acid	HMDB0000812	0.5710	0.0000
tetrahydrodeoxycorticosterone	HMDB0000879	0.4252	0.0000
thromboxane B2	HMDB0003252	0.6593	0.0000
aminoadipic acid	HMDB0000510	0.5776	0.0000
deoxyuridine	HMDB0000012	0.4520	0.0000
S-3-oxodecanoyl cysteamine	HMDB0059773	0.6553	0.0000
glutamine	HMDB0000641	0.4289	0.0000
3-hydroxydecanedioic acid	HMDB0340579	0.4524	0.0000
L-arginine	HMDB0000517	0.2097	0.0111
dihyroxy-1H-indole glucuronide I	HMDB0059997	0.5758	0.0000
11-trans-LTE4	HMDB0062286	0.4089	0.0000
p-cresol glucuronide	HMDB0011686	0.3585	0.0000

Abbreviations: *—Spearman rank correlation coefficient; R—correlation coefficient.

**Table 4:** Negative association between the intake of selected nutrients involved in iAs metabolism and putatively annotated metabolites.

Correlation between Nutrient Intake *** and Metabolites	Both Groups		WL		WH	
	R	*p*	R	*p*	R	*p*
methionine and argininosuccinic acid	NS		−0.3215 **	0.0055	NS	
vitamin B_2_ and argininosuccinic acid	NS		−0.3099 **	0.0076	NS	
vitamin B_12_ and argininosuccinic acid	NS		−0.3039 **	0.0090	NS	
folate and 5-hydroxy-L-tryptophan	NS		NS		−0.2328 **	0.0475
zinc and 5-hydroxy-L-tryptophan	NS		−0.2434 **	0.0380	NS	
vitamin B_2_ and dihyroxy-1H-indole glucuronide I	NS		NS		−0.2568 *	0.0280
vitamin B_6_ and dihyroxy-1H-indole glucuronide I	NS		NS		−0.2489 **	0.0337
vitamin B_2_ and 11-trans-LTE4	NS		−0.2457 **	0.0361	NS	
folate and 11-trans-LTE4	NS		−0.3060 **	0.0085	NS	
zinc and 11-trans-LTE4	−0.1885 **	0.0227	−0.2530 **	0.0308	−0.2486 *	0.0340
methionine and isovalerylglucuronide	NS		NS		−0.2428 **	0.0385
vitamin B_2_ and isovalerylglucuronide	NS		NS		−0.2983 *	0.0100
vitamin B_6_ and isovalerylglucuronide	NS		NS		−0.2812 **	0.0160
folate and isovalerylglucuronide	NS		NS		−0.3754 **	0.0011
zinc and isovalerylglucuronide	NS		NS		−0.2947 *	0.0110
vitamin B_2_ and mevalonic acid	NS		−0.2395 **	0.0413	NS	
vitamin B_6_ and mevalonic acid	NS		−0.2340 *	0.0460	NS	
folate and mevalonic acid	NS		−0.2694 **	0.0212	NS	
vitamin B_6_ and 18-oxocortisol	NS		NS		−0.3558 **	0.0020
methionine and aminoadipic acid	NS		−0.2366 *	0.0440	NS	
vitamin B_2_ and aminoadipic acid	NS		−0.2943 **	0.0115	NS	
vitamin B_12_ and aminoadipic acid	NS		−0.3151 **	0.0066	NS	
folate and aminoadipic acid	NS		−0.2398 **	0.0410	NS	
vitamin B_2_ and 3-hydroxydecanedioic acid	NS		NS		−0.2772 *	0.0180
vitamin B_6_ and S-3-oxodecanoyl cysteamine	NS		NS		−0.3378 **	0.0035
methionine and L-arginine	−0.1834 **	0.0267	NS		−0.3256 **	0.0049
vitamin B_6_ and L-arginine	NS		NS		−0.2451 **	0.0366
zinc and L-arginine	NS		NS		−0.2623 **	0.0250
methionine and p-cresol glucuronide	NS		NS		−0.3876 **	0.0007
vitamin B_12_ and p-cresol glucuronide	NS		NS		−0.3306 **	0.0043
zinc and p-cresol glucuronide	NS		NS		−0.3382 **	0.0034
methionine and thromboxane B2	NS		NS		−0.2606 **	0.0260

Abbreviations: *—Pearson correlation coefficient; **—Spearman rank correlation coefficient; ***—intake of nutrients calculated per kg of body mass; NS—not statistically significant; R—correlation coefficient; *p*—*p*-value; WL—the group of women with lower total urinary arsenic; WH—the group of women with higher total urinary arsenic.

**Table 5:** Positive association between the intake of selected nutrients involved in iAs metabolism and putatively annotated metabolites.

Correlation between Nutrient Intake *** and Metabolites	Both Groups		WL		WH	
	R	*p*	R	*p*	R	*p*
methionine and inosine	0.2227 **	0.0069	NS		0.3426 **	0.0030
vitamin B_2_ and inosine	0.2281 **	0.0056	0.2731 **	0.0194	0.2551 *	0.0290
vitamin B_6_ and inosine	NS		0.2330 **	0.0472	NS	
vitamin B_12_ and inosine	0.1785 **	0.0311	NS		NS	
folate and inosine	0.1770 **	0.0326	NS		NS	
zinc and inosine	0.1637 **	0.0483	NS		NS	
vitamin B_2_ and N-acetyl-L-aspartic acid	NS		NS		0.3387 **	0.0034
vitamin B_6_ and N-acetyl-L-aspartic acid	0.1693 **	0.0411	NS		0.3303 **	0.0043
folate and N-acetyl-L-aspartic acid	NS		NS		0.2839 **	0.0149
zinc and N-acetyl-L-aspartic acid	NS		NS		0.2362 **	0.0442
methionine and tetrahydrodeoxycorticosterone	NS		NS		0.3372 **	0.0035
vitamin B_2_ and tetrahydrodeoxycorticosterone	NS		NS		0.2508 *	0.0320
vitamin B_12_ and tetrahydrodeoxycorticosterone	NS		NS		0.2617 **	0.0253
methionine and deoxyuridine	0.1728 **	0.0222	0.2919 **	0.0122	NS	
vitamin B_6_ and deoxyuridine	0.2563 **	0.0018	0.2549 **	0.0295	NS	
methionine and glutamine	NS		0.2995 **	0.0100	NS	
vitamin B_2_ and glutamine	NS		0.2816 **	0.0158	NS	
vitamin B_6_ and glutamine	0.1994 **	0.0158	0.2563 **	0.0286	NS	
vitamin B_12_ and glutamine	0.2010 **	0.0150	0.3361 **	0.0036	NS	
folate and glutamine	0.2042 **	0.0134	0.4151 **	0.0003	NS	
zinc and glutamine	NS		0.3369 **	0.0036	NS	

Abbreviations: *—Pearson correlation coefficient; **—Spearman rank correlation coefficient; ***—intake of nutrients calculated per kg of body weight; NS—not statistically significant; R—correlation coefficient; *p*—*p*-value; WL—the group of women with lower total urinary arsenic; WH—the group of women with higher total urinary arsenic.

## Data Availability

The data presented in this study are available on request from the corresponding author. The data are not publicly available due to privacy or ethical restrictions.

## Supplementary Materials

The following supporting information can be downloaded at: https://www.mdpi.com/article/10.3390/metabo14010075/s1, Figure S1. Flow chart of urine sample preparation for untargeted metabolomics; Table S1. Reagents used for the metabolomics analysis; Table S2. Dietary intake of selected nutrients involved in iAs metabolism regarding the EAR norm; Table S3. Putatively annotated metabolites.
