# Supplementary material for: Analysis of Relationships between Metabolic Changes and Selected Nutrient Intake in Women Environmentally Exposed to Arsenic
Source: Metabolites. 2024 Jan 22;14(1):75. doi: 10.3390/metabo14010075 (PMC10820439; doi:10.3390/metabo14010075)
Supplement: Supplementary file 1 [file metabolites-14-00075-s001.zip › metabolites-2781771-supplementary.pdf]

1 300 μL solvent was added to 100 μL of urine (thawed at 4°C):

- solvent for assay 1 - ice-cold water and methanol at a 50:50 ratio with internal standards benzoyl-D5 and L-phenylalanine 3,3-D2

- solvent for assay 2 - acetonitrile and methanol 50:50 with the same internal standards

2 vortex for 2 minutes

3 centrifuge at 20,879 rpm for 20 minutes at 4°C

4 supernatants (200 μL) were aliquoted into vials

Figure S1. Flow chart of urine sample preparation for untargeted metabolomics.

Table S1. Reagents used for the metabolomics analysis.

REAGENT	SOURCE	IDENTIFIER
LC-MS-grade methanol	Avantor Performance Materials Poland. S.A.	CAS: 67-56-1
LC-MS-grade acetonitrile	Avantor Performance Materials Poland. S.A.	CAS: 75-05-8
Formic acid 98-100%	CHEM-LAB NV	CAS: 64-18-6
Ammonium formate > 99.995% trace metals basis	Sigma Aldrich	CAS: 540-69-2
Glycine, N-Benzyol (Hippuric acid) (Benzyol-D5, 98%)	Cambridge Isotope Laboratories, Inc.	CAS: 53518-98-2
L-phenylalanine (3,3-D2 98%)	Cambridge Isotope Laboratories, Inc.	CAS: 221346-31-2
Sodium formate	Waters	prepared according to the manufacturer's specifications
Leucine encephalin lock mass solution	Waters	prepared according to the manufacturer's specifications
Ultra-high-purity water	R5 UV Hydrolab system (Wislina, Poland)	-

Table S2. Dietary intake of selected nutrients involved in iAs metabolism regarding the EAR norm.

**Dietary Intake**	**Both Groups**		**WL**		WH		***p*- Value ****	**EAR**
	***n* = 146**		***n* = 73**		***n* = 73**			
	Mean ± SD	Median (Minimum-Maximum)	Mean ± SD	Median (Minimum-Maximum)	Mean ± SD	Median (Minimum-Maximum)		
Methionine (mg/day)	1495.25 ± 464.14	1459.32 (670.33-3832.17)	1441.06 ± 396.79*	1384.17 (670.33-2493.09)	1549.43 ± 520.05	1525.82 (714.21-3832.17)	0.2265	—
Vitamin B_2 (mg/day)	1.33 ± 0.37	1.29 (0.68-2.64)	1.28 ± 0.36	1.23 (0.68-2.64)	1.39 ± 0.38	1.32 (0.77-2.36)	0.0872	0.9
Vitamin B_6 (mg/day)	1.57 ± 0.50	1.57 (0.46-3.46)	1.43 ± 0.45*	1.38 (0.46-2.55)	1.70 ± 0.51	1.63 (0.77-3.46)	0.0018	1.1 (19-50 y)<br>1.3 (>50 y)
Vitamin B_{12} (µg/day)	2.32 ± 1.41	2.05 (0.57-13.39)	2.09 ± 0.81	2.00 (0.57-4.92)	2.54 ± 1.80	2.07 (0.57-13.39)	0.3221	2.0
Folate (µg/day)	260.46 ± 92.46	242.46 (78.14-682.02)	250.70 ± 93.97	232.37 (104.70-682.02)	270.22 ± 90.51	250.49 (78.14-618.13)	0.0809	320.0
Zinc (mg/day)	8.19 ± 2.03	8.10 (4.57-13.22)	8.05 ± 2.08*	7.89 (4.57-13.22)	8.33 ± 1.98*	8.29 (4.65-12.66)	0.4069	6.8

Abbreviations: * — parametric distribution data (used Shapiro-Wilk test $p \leq 0.05$); ** — the Student's t-test (for parametric distribution) and Mann–Whitney U test (for nonparametric distribution) were used to examine differences between WL and WH; EAR – estimated average requirement for Polish population; WL—the group of women with lower total urinary arsenic; WH—the group of women with higher total urinary arsenic.

Table S3. Putatively annotated metabolites.

Number	Column/ESI mode	m/z	r.t	Calculated molecular weight (Da)	Delta (ppm)#	Monoisotopic molecular weight (Da)	Adduct	Annotated compounds name (ID in HMDB)	Fit (%)	Subpathway	Chemical Taxonomy - Class	*p* -Value	Test	WL vs. WH
1	BEH Amide (+)	197.0809	4.61	196.0736	-0.3	196.0736	M+H	Dihydroferulic acid (HMDB0062121)	0.94	Food	Phenylpropanoic acids	0.0000	Mann–Whitney U test	in the WH higher signal intensity
2	BEH Amide (+)	287.1602	5.43	254.1267	-0.2	254.1267	M+CH3OH+H	Pyrraline (HMDB0033143)	0.95	Food	Carboxylic acids and derivatives	0.0000	Mann–Whitney U test	in the WH higher signal intensity
3	BEH Amide (+)	291.1300	8.27	290.1227	-0.3	290.1226	M+H	Argininosuccinic acid (HMDB0000052)	0.98	Alanine, aspartate and glutamate metabolism; Arginine biosynthesis	Carboxylic acids and derivatives	0.0000	Mann–Whitney U test	in the WH higher signal intensity
4	BEH Amide (+)	138.0553	4.35	137.0480	-2.5	137.0477	M+H	Trigonelline (HMDB0000875)	0.84	Food	Not Available	0.0000	Mann–Whitney U test	in the WH higher signal intensity
5	BEH Amide (+)	179.0052	5.56	177.9979	-2.4	177.9975	M+H	Arsenobetaine (HMDB0033206)	0.88	Food	Organometalloid compounds	0.0000	Mann–Whitney U test	in the WH higher signal intensity
6	BEH Amide (+)	203.0817	5.73	220.0844	1.8	220.0848	M+H-H2O	5-Hydroxy-L-tryptophan (HMDB0000472)	0.86	Tryptophan metabolism	Indoles and derivatives	0.0000	Mann–Whitney U test	in the WH higher signal intensity
7	BEH Amide (+)	278.1236	7.23	278.1009	-2.6	278.1002	M+NH4-H2O	Isovalerylglucuronide (HMDB0002091)	0.95	Starch and Sucrose Metabolism	Organooxygen compounds	0.0000	Mann–Whitney U test	in the WH higher signal intensity
8	BEH Amide (-)	147.0659	3.72	148.0732	2.4	148.0736	M-H	Mevalonic acid (HMDB0000227)	0.88	Terpenoid backbone biosynthesis	Fatty Acyls	0.0000	Mann–Whitney U test	in the WH higher signal intensity
9	BEH Amide (-)	212.0116	2.05	175.0630	1.9	175.0633	M+K-2H	Indoleacetic acid (HMDB0000197)	0.88	Food	Indoles and derivatives	0.0000	Mann–Whitney U test	in the WH higher signal intensity
10	BEH Amide (-)	217.1440	0.97	172.1458	3.1	172.1463	M+FA-H	Capric acid (HMDB0000511)	0.92	Food	Fatty Acyls	0.0000	Mann–Whitney U test	in the WH higher signal intensity
11	BEH Amide (-)	267.0732	2.59	268.0805	1.0	268.0808	M-H	Inosine (HMDB0000195)	0.99	Purine metabolism	Purine nucleosides	0.0137	Mann–Whitney U test	in the WH lower signal intensity
12	HSST3 (+)	287.2372	6.68	304.2399	1.1	304.2402	M+H-H2O	Arachidonic acid (HMDB0001043)	0.99	Food	Fatty Acyls	0.0000	Mann–Whitney U test	in the WH higher signal intensity
13	HSST3 (+)	341.1743	6.28	376.1870	4.2	376.1886	M+H-H2O	18-Oxocortisol (HMDB0000332)	1.00	Steroidogenic pathway	Steroids and steroid derivatives	0.0000	Mann–Whitney U test	in the WH higher signal intensity
14	HSST3 (+)	492.2192	6.78	459.1857	2.0	459.1866	M+CH3OH+H	5-Methyltetrahydrofolic acid (HMDB0001396)	0.97	Food	Pteridines and derivatives	0.0000	Mann–Whitney U test	in the WH higher signal intensity
15	HSST3 (+)	139.0030	2.07	174.0157	4.2	174.0164	M+H-2H2O	Dehydroascorbic acid (HMDB0001264)	0.94	Food	Lactones	0.0000	Mann–Whitney U test	in the WH higher signal intensity
16	HSST3 (+)	179.0052	0.92	177.9979	-2.4	177.9975	M+H	Arsenobetaine (HMDB0033206)	0.88	Food	Organometalloid compounds	0.0000	Student's *t*-test	in the WH higher signal intensity
17	HSST3 (+)	318.1658	3.73	276.1319	0.9	276.1321	M+ACN+H	Saccharopine (HMDB0000279)	0.94	Food	Carboxylic acids and derivatives	0.0000	Mann–Whitney U test	in the WH higher signal intensity
18	HSST3 (+)	370.0956	5.43	352.0618	-0.3	352.0617	M+NH4	3'-O-Methyl-(-)-epicatechin-5-O-sulphate (HMDB0029176)	0.98	Food	Flavonoids	0.0000	Mann–Whitney U test	in the WH higher signal intensity
19	HSST3 (+)	198.0376	1.09	175.0484	-1.8	175.0481	M+Na	N-Acetyl-L-aspartic acid (HMDB0000812)	0.82	Alanine, aspartate and glutamate metabolism	Carboxylic acids and derivatives	0.0000	Mann–Whitney U test	in the WH higher signal intensity
20	HSST3 (+)	335.2576	6.82	334.2503	1.4	334.2508	[M+H]+	Tetrahydrodeoxycorticosterone (HMDB0000879)	0.99	Steroid hormone biosynthesis	Steroids and steroid derivatives	0.0000	Mann–Whitney U test	in the WH higher signal intensity
21	HSST3 (+)	388.2690	6.69	370.2352	1.0	370.2355	M+NH4	Thromboxane B2 (HMDB0003252)	1.00	Arachidonic acid metabolism	Fatty Acyls	0.0000	Student's *t*-test	in the WH higher signal intensity
22	HSST3 (+)	133.0762	2.88	150.0789	2.8	150.0793	M+H-H2O	6-Methylnicotinamide (HMDB0013704)	0.81	Food	Pyridines and derivatives	0.0000	Mann–Whitney U test	in the WH higher signal intensity
23	HSST3 (+)	379.1388	6.03	378.1315	-0.1	378.1315	M+H	3,4-DHPEA-EA (HMDB0029304)	0.96	Food	Prenol lipids	0.0000	Mann–Whitney U test	in the WH higher signal intensity
24	HSST3 (+)	381.1181	6.71	398.1208	1.2	398.1213	M+H-H2O	5-(3',4'-dihydroxyphenyl)-gamma-valerolactone-3'-O-glucuronide (HMDB0029190)	1.00	Food	Phenol ethers	0.0000	Mann–Whitney U test	in the WH higher signal intensity
25	HSST3 (-)	145.0502	5.53	164.0686	-0.7	164.0685	M-H2O-H	L-Fucose (HMDB0000174)	0.95	Food	Organooxygen compounds	0.0000	Mann–Whitney U test	in the WH higher signal intensity
26	HSST3 (-)	160.0612	1.57	161.0685	1.9	161.0688	M-H	Aminoadipic acid (HMDB0000510)	0.91	Lysine degradation	Carboxylic acids and derivatives	0.0000	Student's *t*-test	in the WH higher signal intensity
27	HSST3 (-)	164.0351	3.43	165.0424	1.2	165.0426	M-H	6-Methoxy-2(3H)-benzoxazolone (HMDB0036582)	0.75	Food	Benzoxazoles	0.0000	Student's *t*-test	in the WH higher signal intensity
28	HSST3 (-)	227.0669	3.64	228.0742	1.8	228.0746	M-H	Deoxyuridine (HMDB0000012)	0.89	Pyrimidine metabolism	Pyrimidine nucleosides	0.0000	Mann–Whitney U test	in the WH higher signal intensity
29	HSST3 (-)	290.1425	6.71	245.1443	2.7	245.1449	M+FA-H	S-3-oxodecanoyl cysteamine (HMDB0059773)	0.96	Linoleic acid metabolism	Fatty Acyls	0.0000	Mann–Whitney U test	in the WH higher signal intensity
30	HSST3 (-)	301.0724	6.54	256.0742	-2.5	256.0736	M+FA-H	2-Dehydro-O-desmethylangolensin (HMDB0041647)	0.92	Food	Stilbenes	0.0000	Mann–Whitney U test	in the WH higher signal intensity
31	HSST3 (-)	127.0514	3.50	146.0698	-4.4	146.0691	M-H2O-H	Glutamine (HMDB0000641)	0.92	D-Glutamine and D-glutamate metabolism; Arginine biosynthesis; Alanine, aspartate and glutamate metabolism; Aminoacyl-tRNA biosynthesis; Nitrogen metabolism; Pyrimidine metabolism; Purine metabolism; Glyoxylate and dicarboxylate metabolism	Carboxylic acids and derivatives	0.0000	Mann–Whitney U test	in the WH higher signal intensity
32	HSST3 (-)	157.0867	4.90	158.0940	1.9	158.0943	M-H	trans-4-Hydroxycyclohexylacetic acid (HMDB0000909)	0.84	Food	Organooxygen compounds	0.0000	Mann–Whitney U test	in the WH higher signal intensity
33	HSST3 (-)	199.0971	5.17	218.1155	-0.3	218.1154	M-H2O-H	3-hydroxydecanedioic acid (HMDB0340579)	0.91	Fatty acids metabolism	Hydroxy acids and derivatives	0.0000	Mann–Whitney U test	in the WH higher signal intensity
34	HSST3 (-)	211.0608	5.45	174.1122	-3.0	174.1117	M+K-2H	L-Arginine (HMDB0000517)	0.89	Arginine biosynthesis; Arginine and proline metabolism; Aminoacyl-tRNA biosynthesis	Carboxylic acids and derivatives	0.0001	Mann–Whitney U test	in the WH higher signal intensity
35	HSST3 (-)	306.0628	3.67	325.0812	-4.4	325.0798	M-H2O-H	Dihyroxy-1H-indole glucuronide I (HMDB0059997)	0.83	Modified metabolites with glucuronidation	Organooxygen compounds	0.0000	Mann–Whitney U test	in the WH higher signal intensity
36	HSST3 (-)	438.2328	6.93	439.2401	-1.9	439.2392	M-H	11-trans-LTE4 (HMDB0062286)	0.99	Arachidonic acid metabolism	Fatty Acyls	0.0000	Mann–Whitney U test	in the WH higher signal intensity
37	HSST3 (-)	165.0918	6.91	166.0991	1.7	166.0994	M-H	2,4,7-Decatrienoic acid (HMDB0035235)	0.75	Food	Fatty Acyls	0.0000	Mann–Whitney U test	in the WH higher signal intensity
38	HSST3 (-)	193.1230	7.29	194.1303	2.0	194.1307	M-H	Ethyl (2E,4Z,7Z)-Decatrienoate (HMDB0035376)	0.92	Food	Fatty Acyls	0.0000	Student's *t*-test	in the WH higher signal intensity
39	HSST3 (-)	213.0764	5.17	168.0782	2.6	168.0786	M+FA-H	(4-Hydroxy-3-methoxyphenyl)ethanol (HMDB0038925)	0.94	Food	Carboxylic acids and derivatives	0.0000	Mann–Whitney U test	in the WH higher signal intensity
40	HSST3 (-)	259.0755	3.50	214.0773	1.4	214.0776	M+FA-H	Methyl bisnorbiotinyl ketone (HMDB0004822)	0.98	Food	Thienoimidazolidines	0.0000	Mann–Whitney U test	in the WH higher signal intensity
41	HSST3 (-)	265.0714	5.93	284.0898	-0.7	284.0896	M-H2O-H	p-Cresol glucuronide (HMDB0011686)	0.84	Tyrosine metabolism	Organooxygen compounds	0.0000	Mann–Whitney U test	in the WH higher signal intensity

Abbreviations: m/z—mass-to-charge ratio; r.t—retention time; WL—the group of women with lower total urinary arsenic; WH—the group of women with higher total urinary arsenic; # - delta between calculated molecular weight and monoisotopic molecular weight.
